# Real-world antipsychotic prescribing patterns and mortality in schizophrenia: a registry cohort study from China

**DOI:** 10.1017/S204579602610078X

**Published:** 2026-08-04

**Authors:** Hongbo He, Lingyan Dai, Sicong Ma, Fu-Jun Jia, Bin Chen, Haicheng Lin, Shi-Bin Wang, Wenyan Tan, Jie Li

**Affiliations:** 1 Guangdong Mental Health Center, Guangdong Provincial People’s Hospital, Guangdong Academy of Medical Sciences, Southern Medical University, Guangzhou, China; 2 Global Health Research Center, Guangdong Provincial People’s Hospital, Guangdong Academy of Medical Sciences, Southern Medical University, Guangzhou, China; 3 School of Public Health, Southern Medical University, Guangzhou, China

**Keywords:** antipsychotic agents, China, mortality, real-world, schizophrenia

## Abstract

**Aims:**

Schizophrenia is a chronic psychiatric disorder associated with significantly elevated mortality. While antipsychotics are the cornerstone of treatment, their long-term effects on survival remain uncertain, particularly in non-Western populations. We aimed to assess the real-world association between antipsychotic treatment patterns and all-cause mortality among adults with schizophrenia in China.

**Methods:**

This population-based cohort study included 435,816 adults from the Community Schizophrenia Registry System of Guangdong Province, China. Patients were classified into four groups: antipsychotic monotherapy, polytherapy, non-antipsychotic treatment and non-drug treatment. Cox proportional hazards models were used to estimate adjusted hazard ratios (aHRs) for mortality.

**Results:**

Over a median follow-up of 7.2 years, 73,527 deaths (16.9%) occurred. Antipsychotic polytherapy (57.6%) was the most common regimen, followed by monotherapy (27.6%). Compared with the non-drug group, mortality risks were significantly lower for polytherapy (aHR: 0.18; 95% CI: 0.18–0.19), monotherapy (aHR: 0.24; 95% CI: 0.24–0.25) and non-antipsychotic treatment (aHR: 0.22; 95% CI: 0.21–0.23). Both first- and second-generation antipsychotics showed comparable mortality benefits. While most polytherapy regimens performed similarly to or better than monotherapy, combinations like risperidone–sulpiride were associated with higher mortality. Subgroup analyses showed stronger benefits in women, younger individuals, rural residents and those with less severe illness.

**Conclusions:**

Antipsychotic treatment is associated with significantly reduced mortality among patients with schizophrenia in China, with effects varying by patient characteristics and drug regimen.

Summary box **What is already known on this topic**

Antipsychotic medications are the mainstay of schizophrenia treatment and have been linked to both reduced psychiatric relapse and increased physical health risks. However, most evidence on their long-term impact on mortality comes from Western populations, with limited real-world data from China or other non-Western settings.

**What this study adds**

This large population-based study shows that both monotherapy and polytherapy with antipsychotics are associated with substantially reduced all-cause mortality in Chinese patients with schizophrenia. Mortality benefits were consistent across first- and second-generation antipsychotics and were more pronounced in certain subgroups, such as women and rural residents.

**How this study might affect research, practice or policy**

These findings provide robust real-world evidence supporting antipsychotic use in reducing mortality among schizophrenia patients. They highlight the need for individualized treatment strategies and may inform future clinical guidelines and mental health policy in China and similar settings.

## Introduction

Schizophrenia is a chronic, severe mental disorder affecting roughly 1% of the population (Freedman, 2003). Patients with schizophrenia suffer markedly higher mortality rates than the general population, translating to an approximately 10–20-year reduction in average life (Liu *et al.*, 2017). Alarmingly, recent studies indicate that the mortality gap between individuals with schizophrenia and the general public has not improved – it may even be widening over time (Rosenbaum, 2016). This premature mortality contributes substantially to the public health burden of schizophrenia, which is already among the leading causes of disability in China (He *et al.*, 2020). Clearly, a stronger evidence base is needed to understand and address the factors driving excess mortality in this population.

A large proportion of the excess deaths in schizophrenia are due to natural causes rather than unnatural causes (Olfson *et al.*, 2015). While suicide and accidents account for part of the premature mortality, about two-thirds of excess deaths are attributed to natural causes such as cardiovascular disease, diabetes, respiratory illness and other chronic medical conditions (Walker *et al.*, 2015). Underlying factors like unhealthy lifestyles and the side effects of antipsychotic medications are thought to contribute to this elevated risk (Laursen *et al.*, 2014). Antipsychotic drugs are the cornerstone of schizophrenia treatment and have proven efficacy in reducing relapse and stabilizing patients over the long term (Leucht *et al.*, 2012a, 2012b). For many patients, continuous antipsychotic therapy substantially improves symptom control and may prevent life-threatening events such as suicide or extreme behavioural disturbances (Johnsen *et al.*, 2020; Leucht *et al.*, 2024). However, long-term use of antipsychotics is also associated with metabolic side effects (weight gain, dyslipidaemia, diabetes) and cardiovascular complications, which can increase the risk of mortality from physical illnesses (Burschinski *et al.*, 2023). The net impact of prolonged antipsychotic treatment on survival has been a subject of ongoing debate.

Notably, most evidence on schizophrenia outcomes to date comes from Western countries or from controlled clinical trials, with relatively little data drawn from real-world settings in China (Schneider-Thoma *et al.*, 2018; Taipale *et al.*, 2020; Tiihonen *et al.*, 2022; Efthimiou *et al.*, 2024). Clinical trials typically have strict inclusion criteria and may not capture the full spectrum of patients seen in routine practice, limiting their generalizability (Sherman *et al.*, 2016; Tiihonen *et al.*, 2017). Moreover, China differs from Western contexts in terms of genetic background, healthcare infrastructure and lifestyle factors, which could influence both treatment patterns and health outcomes (Solmi *et al.*, 2024). Western mortality estimates for schizophrenia may therefore not be directly applicable to Chinese patients.

In recent years, the Chinese government has implemented a national management programme for severe mental disorders (often referred to as the ‘686 Program’) (Liu *et al.*, 2011), which has enabled the creation of extensive regional psychiatric registries. Here, we leverage the Guangdong Province Severe Mental Disorders Management Database, a comprehensive psychiatric case registry in southern China, including approximately 450,000 patients with schizophrenia. In the current study, we aimed to characterize the patterns of antipsychotic treatment in this population – including the use of first- vs. second-generation antipsychotics, monotherapy vs. polytherapy and other prescribing trends – and to evaluate the association between these treatment patterns and subsequent mortality risk. Through this investigation, we sought to strengthen the empirical foundation for managing schizophrenia in China and to inform clinical and public health strategies to reduce premature mortality in this vulnerable population.

## Methods

### Data source

This study was a retrospective cohort analysis based on the Community Schizophrenia Registry System of Guangdong Province, China. This province-wide registry systematically collects and manages clinical data on individuals diagnosed with schizophrenia across all 21 prefecture-level cities in Guangdong. It serves as a unified information platform for the ongoing monitoring, treatment management and follow-up of patients with schizophrenia in community settings. The registry contains standardized and prospectively maintained records, including demographic information, clinical diagnoses (coded according to ICD-10 criteria), treatment regimens, follow-up encounters and vital status. Data are regularly updated through outpatient visits, home follow-ups and telephone interviews, ensuring both timeliness and completeness. Quality control is enforced at the municipal and provincial levels through routine data audits, logic checks and standardized reporting protocols. As one of the largest regional schizophrenia databases in China, the registry currently includes approximately 450,000 patients, providing a robust real-world population base for longitudinal research. For the present study, data from January 2000 to March 2024 were extracted, focusing specifically on individuals diagnosed with schizophrenia (ICD-10 code F20.x).

### Study population

The study population consisted of adults diagnosed with schizophrenia who were enrolled in the Guangdong Province registry during the study period and met predefined eligibility criteria. Inclusion required a documented diagnosis of schizophrenia in the registry and at least one follow-up assessment to ascertain treatment status and survival. As of March 2024, 450,242 individuals had been registered in the database. We excluded individuals with missing key information on treatment or outcomes at baseline, as well as a small number of implausible or duplicate records (e.g., obvious data entry errors in dates or age) (n = 4,154), and those aged under 18 years (n = 12,272). After applying these criteria, a total of 435,816 adult patients were included in this study. All patients were alive at the time of cohort entry and were followed forward for mortality outcomes. The study was approved by the institutional review board of the Guangdong Provincial People’s Hospital, with a waiver of informed consent because the research utilized de-identified registry data.

### Exposure

The exposure of interest was the antipsychotic treatment regimen, as recorded in the registry’s medication records. Treatment exposure was defined based on medication patterns observed across the follow-up period, reflecting cumulative real-world prescribing trajectories rather than a fixed baseline exposure. At each routine follow-up visit in the registry, psychiatrists document the medications the patient is receiving. The study antipsychotics comprised both oral and injectable formulations, encompassing first-generation antipsychotics (FGAs, e.g., perphenazine, sulpiride, chlorpromazine, penfluridol), second-generation antipsychotics (SGAs, e.g., risperidone, olanzapine, clozapine, quetiapine, aripiprazole, amisulpride) and long-acting injectables (LAIs, e.g., paliperidone LAI, haloperidol LAI, perphenazine LAI, risperidone LAI). A detailed listing of medications is provided in Supplementary Table 1.

Patients were classified into one of four mutually exclusive groups according to their psychotropic medication status: (1) Antipsychotic monotherapy, defined as receiving only one type of antipsychotic throughout the entire follow-up period; (2) Antipsychotic polytherapy, defined as receiving two or more antipsychotics, either concurrently (combination therapy) or sequentially (switching therapy); (3) Non-antipsychotic treatment, defined as not receiving any antipsychotic medication (e.g., hypnotics, antihyperglycemics, antidementia drugs, anticholinergics, antiepileptics, antihypertensives, anxiolytics, antimicrobials, anticoagulants, antiparkinsonians, antidepressants and antimanic agents), though the patient may have been receiving other non-antipsychotic psychotropic treatments or psychosocial interventions; and (4) Non-drug treatment, defined as receiving no psychotropic medications of any kind (completely untreated with medication).

### Outcome

The primary outcome of this study was all-cause mortality during follow-up. Deaths from any cause were identified through the registry’s surveillance system, primarily based on reports from patients’ family members during routine follow-up. Although the registry is not yet linked to the provincial Center for Disease Control and Prevention mortality database, community-based mental health practitioners play a crucial role in verifying death information. When a death is reported, they routinely cross-check the information through multiple sources, including neighbourhood or village committees and staff at community health centres responsible for local mortality surveillance. This multi-source verification process ensures a high level of data accuracy and reliability.

Follow-up for each patient began at the date of cohort entry and continued until the occurrence of death or the end of the study period, whichever came first. Survivors were censored on 30 March 2024, the final date of follow-up. In this system, loss of follow-up was minimal, due to the structured management protocols, mandatory reporting responsibilities and strong linkage between community-based psychiatric care and public health infrastructure. Patients who relocated outside the province or could not be contacted were censored on the date of their last confirmed follow-up with verified survival status.

### Covariates

We extracted a range of covariates from the registry to control for potential confounding factors, such as demographic characteristics, comorbid medical conditions and indicators of schizophrenia severity. Demographic variables included sex, age at schizophrenia diagnosis, age at cohort entry, ethnicity (Han vs. minority), educational attainment (categorized as illiterate, primary, secondary, higher or college and above), marital status (married vs. unmarried), poverty level (low vs. high, according to government-defined thresholds of annual income) and residence (urban vs. rural). Comorbid conditions included documented diagnoses of malignant neoplasms, hypertension, stroke, coronary heart disease, chronic obstructive pulmonary disease, diabetes, hepatitis, tuberculosis, arrhythmia, other infectious diseases (classified as physical comorbidities), as well as additional psychiatric disorders (classified as psychiatric comorbidities). Severity of schizophrenia was assessed using recorded indicators, such as frequency of suicide attempts or self-harm episodes during follow-up, presence of positive and negative symptoms and receipt of intensive mental health management.

## Statistical analysis

Descriptive analyses were first performed to characterize the cohort and compare baseline variables according to the antipsychotic groups. Continuous variables were presented as medians with interquartile ranges (IQR), and categorical variables were described using frequencies and percentages. Frequency distributions and temporal trends for each medication were visualized. Polytherapy patterns were illustrated using network diagrams to depict interconnections among medications.

For the main analysis of the association between antipsychotic treatment and mortality, we employed Cox proportional hazards regression models. Time-to-event (survival time) was measured from baseline to the date of death or censoring. We fitted Cox models to estimate hazard ratios (HRs) and 95% confidence intervals (CIs) for all-cause mortality for each treatment category, using the non-drug group as the reference. Multivariable models were adjusted for demographic factors, comorbidities and schizophrenia severity indicators to address potential confounding.

We performed several stratified analyses to examine how the potential effect modifiers affected our findings. In particular, analyses were stratified by sex to examine sex-specific differences in treatment-related mortality. Age-specific analyses were also performed, acknowledging that mortality risk and prescribing patterns may vary with age. Given that baseline illness severity could influence both the choice of receiving polytherapy and the risk of death, we further stratified the analyses by intensive mental health management status serving as a proxy indicator of the severity of schizophrenia. Finally, considering the substantial difference in available medical resources and characteristics of patients between urban and rural areas, we examined these associations by region of residence.

To assess the robustness of findings, three sets of sensitivity analyses were conducted. First, we excluded patients with follow-up durations shorter than 180 days to minimize potential misclassification bias related to short-term medication exposure. Second, given the registry system upgrade in 2018 that improved prescription data completeness, we repeated analyses excluding patients enrolled before 2018 to examine the potential impact of information bias due to incomplete medication records. Third, recognizing that physical and psychiatric comorbidities may influence mortality through distinct pathways, we performed additional analyses that modelled these domains separately. Physical comorbidities included major chronic medical conditions recorded in the registry, while psychiatric comorbidity was defined as the presence of additional psychiatric disorders. These variables were incorporated separately into multivariable models to better account for their differential associations with mortality and treatment patterns.

All statistical analyses were performed using R software, version 4.2.1 (R Foundation for Statistical Computing). Two-tailed *P* values less than 0.05 were considered statistically significant.

## Result

### Patient characteristics at baseline

The study included 435,816 patients diagnosed with schizophrenia, among whom 73,527 (16.9%) died during the follow-up period. At cohort entry, the median age was 43.4 years (IQR: 32.4–54.4 years), with men accounting for 54.1%. Of these patients, 251,223 (57.6%) received antipsychotic polytherapy, 120,487 (27.6%) received antipsychotic monotherapy, 57,335 (13.2%) received no medication (non-drug group) and 6,771 (1.6%) were prescribed only non-antipsychotic drugs. Compared with those on monotherapy and polytherapy, non-drug treatment patients were generally older at schizophrenia diagnosis, had lower educational attainment and socioeconomic status, resided predominantly in rural areas and were less frequently enrolled in intensive mental health management programmes (Table 1).

**Table 1.** Baseline characteristics of schizophrenia patients by antipsychotic group Table 1 long description.

		Antipsychotic group			
Characteristics	Overall	Non-drug	Non-antipsychotic	Monotherapy	Polytherapy
No. of participants	435816	57335	6771	120487	251223
Follow-up time, y, median (IQR)	7.2 [3.6, 11.2]	4.5 [2.5, 7.4]	8.3 [4.8, 11.6]	7.0 [2.7, 11.1]	7.6 [4.5, 11.5]
Death, n (%)					
No	362289 (83.1)	14981 (26.1)	5545 (81.9)	105915 (87.9)	235848 (93.9)
Yes	73527 (16.9)	42354 (73.9)	1226 (18.1)	14572 (12.1)	15375 (6.1)
Sex, n (%)					
Men	235887 (54.1)	33479 (58.4)	3459 (51.1)	63546 (52.7)	135403 (53.9)
Women	199929 (45.9)	23856 (41.6)	3312 (48.9)	56941 (47.3)	115820 (46.1)
Age at schizophrenia diagnosis, y, median (IQR)	29.0 [21.0, 41.0]	35.0 [24.0, 50.0]	35.0 [23.0, 48.0]	32.0 [23.0, 44.0]	27.0 [20.0, 37.0]
Age at cohort entry, y, median (IQR)	43.4 [32.4, 54.4]	54.2 [42.3, 67.5]	50.6 [39.9, 61.9]	46.5 [35.5, 56.8]	39.7 [30.0, 49.8]
Ethnicity, n (%)					
Han	433125 (99.4)	56980 (99.4)	6731 (99.4)	119599 (99.3)	249815 (99.4)
Non-Han	2691 (0.6)	355 (0.6)	40 (0.6)	888 (0.7)	1408 (0.6)
Education attainment, n (%)					
Illiterate or semi-illiterate	67119 (15.4)	16155 (28.2)	1535 (22.7)	19402 (16.1)	30027 (12.0)
Primary school	141969 (32.6)	20189 (35.2)	2407 (35.5)	40031 (33.2)	79342 (31.6)
Junior high school	158701 (36.4)	16730 (29.2)	2008 (29.7)	41820 (34.7)	98143 (39.1)
High school	48667 (11.2)	3238 (5.6)	620 (9.2)	13305 (11.0)	31504 (12.5)
College and over	19360 (4.4)	1023 (1.8)	201 (3.0)	5929 (4.9)	12207 (4.9)
Marital status, n (%)					
Married	219230 (50.3)	29046 (50.7)	3873 (57.2)	64219 (53.3)	122092 (48.6)
Unmarried	216586 (49.7)	28289 (49.3)	2898 (42.8)	56268 (46.7)	129131 (51.4)
Economic status, n (%)					
Low	229314 (52.6)	31570 (55.1)	3437 (50.8)	58496 (48.5)	135811 (54.1)
High	206502 (47.4)	25765 (44.9)	3334 (49.2)	61991 (51.5)	115412 (45.9)
Region of residence, n (%)					
Rural	178048 (40.9)	31938 (55.7)	2845 (42.0)	48392 (40.2)	94873 (37.8)
Urban	257768 (59.1)	25397 (44.3)	3926 (58.0)	72095 (59.8)	156350 (62.2)
No. of comorbid conditions, n (%)					
None	422060 (96.8)	56405 (98.4)	6391 (94.4)	115908 (96.2)	243356 (96.9)
1−2	10874 (2.5)	728 (1.3)	291 (4.3)	3542 (2.9)	6313 (2.5)
≥3	2882 (0.7)	202 (0.4)	89 (1.3)	1037 (0.9)	1554 (0.6)
Frequency of suicide attempts or self-harm episodes, n (%)					
None	410082 (94.1)	55819 (97.4)	6507 (96.1)	113491 (94.2)	234265 (93.2)
1−2	16945 (3.9)	832 (1.5)	154 (2.3)	4769 (4.0)	11190 (4.5)
≥3	8789 (2.0)	684 (1.2)	110 (1.6)	2227 (1.8)	5768 (2.3)
Positive symptoms, n (%)					
No	70035 (16.1)	14354 (25.0)	1080 (16.0)	16786 (13.9)	37815 (15.1)
Yes	365781 (83.9)	42981 (75.0)	5691 (84.0)	103701 (86.1)	213408 (84.9)
Negative symptoms, n (%)					
No	175252 (40.2)	23478 (40.9)	2368 (35.0)	47736 (39.6)	101670 (40.5)
Yes	260564 (59.8)	33857 (59.1)	4403 (65.0)	72751 (60.4)	149553 (59.5)
Intensive mental health management, n (%)					
No	181671 (41.7)	51675 (90.1)	3901 (57.6)	56638 (47.0)	69457 (27.6)
Yes	254145 (58.3)	5660 (9.9)	2870 (42.4)	63849 (53.0)	181766 (72.4)

Continuous variables are described as mean (SD) for those with normal distribution and median (Interquartile Range, IQR) for those with non-normal distribution. Categorical variables are described as number (percent). Non-antipsychotic medications comprise a diverse array of pharmaceuticals, such as hypnotics, antihyperglycemics, antidementia drugs, anticholinergics, antiepileptics, antihypertensives, anxiolytics, antimicrobials, anticoagulants, antiparkinsonians, antidepressants and antimanic agents.

### Patterns of antipsychotic drug utilization

**Figure 1.**
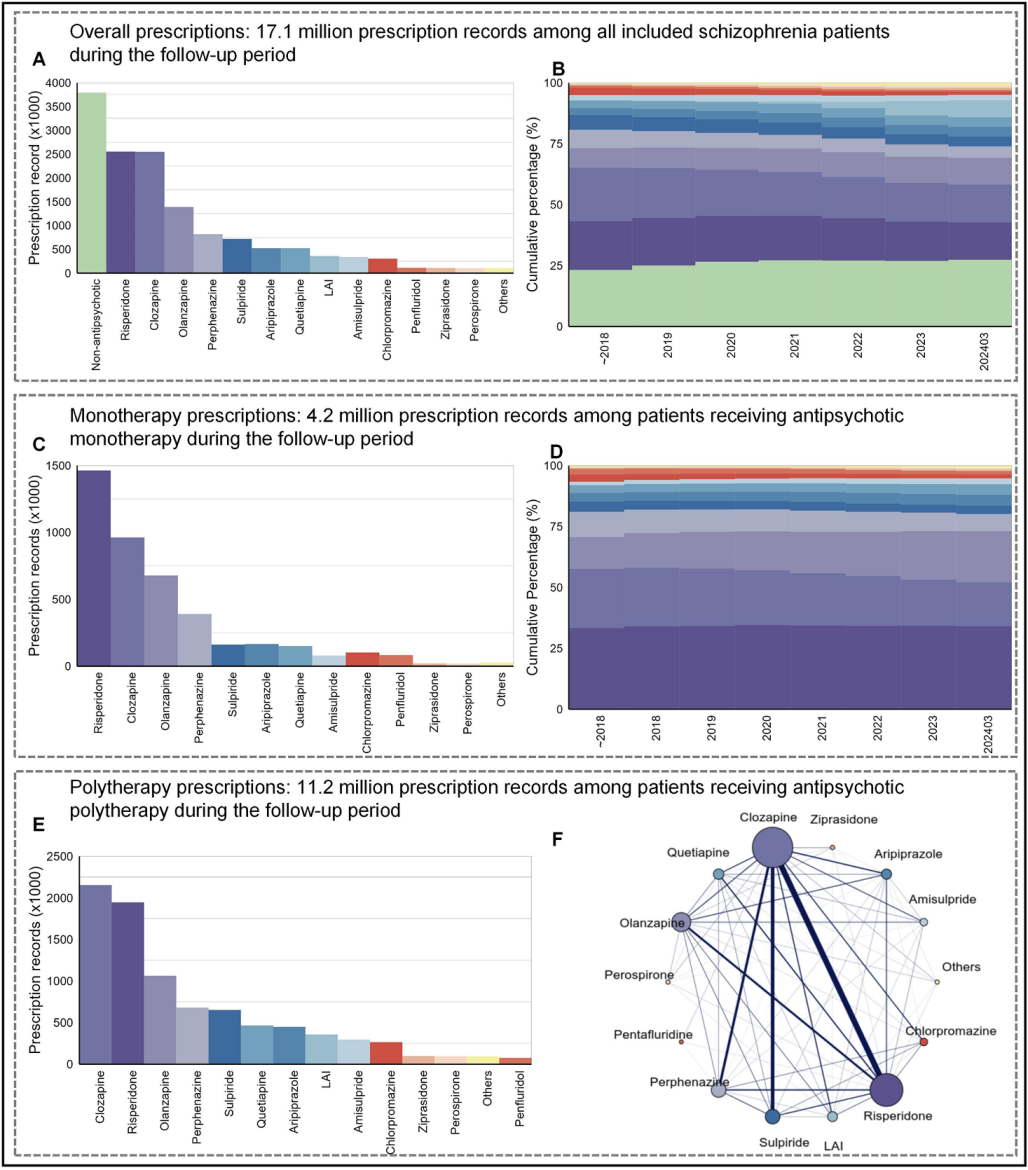
Patterns of antipsychotic treatment among schizophrenia patients. (a) Frequency distribution of specific antipsychotic medications prescribed to schizophrenia patients during follow-up. (b) Trends over time showing annual cumulative percentages for the most frequently prescribed antipsychotics. (c) Frequency distribution of antipsychotic medications prescribed among patients receiving monotherapy. (d) Temporal trends indicating cumulative prescription percentages for antipsychotic monotherapy agents during the study period. (e) Frequency distribution of antipsychotic medications prescribed within polytherapy regimens. (f) Commonly prescribed antipsychotic combinations within polytherapy, highlighting frequently used drug pairs such as clozapine-risperidone, clozapine-sulpiride and clozapine-perphenazine. Figure 1 long description.

Over a median follow-up of 7.2 years (IQR: 3.6–11.2 years), 17.1 million prescription records were generated for the included patients. Risperidone, clozapine and olanzapine were the three most frequently prescribed antipsychotics (Fig. 1a). The use of LAI antipsychotics increased steadily over the study period, despite their overall low prescription frequency compared to oral formulations (Fig. 1a,b). Among oral antipsychotics, the use of olanzapine steadily increased annually, whereas clozapine prescription rates gradually declined (Fig. 1b). In patients receiving monotherapy, risperidone, clozapine and olanzapine remained the most prescribed medications, exhibiting similar temporal trends (Fig. 1c,d). Among polytherapy regimens, clozapine-based combinations were most frequently prescribed, particularly in conjunction with risperidone, sulpiride and perphenazine (Fig. 1e,f).

### Association between antipsychotic treatment and the risk of mortality

The highest mortality rate was observed among the non-drug group (137.82 per 1,000 person-years [PY]). In comparison, mortality rates were significantly lower in the non-antipsychotic (22.44 per 1,000 PY), monotherapy (17.61 per 1,000 PY) and polytherapy groups (7.88 per 1,000 PY). After adjustment for demographic factors, comorbidities and schizophrenia severity, mortality risks significantly decreased in all medication groups relative to the non-drug group: non-antipsychotic (adjusted hazard ratio [aHR]: 0.22; 95% CI: 0.21–0.23), monotherapy (aHR: 0.24; 95% CI: 0.24–0.25) and polytherapy (aHR: 0.18; 95% CI: 0.18–0.19) (Table 2).

**Table 2.** Association between antipsychotic treatment and mortality risk among schizophrenia patients Table 2 long description.

Antipsychotic group	Deaths/1000 PY	Model 1	Model 2	Model 3	P for interaction
**Total participants (N = 435,816)**					
Non-drug	42354/307.31	Reference	Reference	Reference	
Non-antipsychotic	1226/54.64	0.17 (0.16, 0.18)	0.17 (0.16, 0.18)	0.22 (0.21, 0.23)	
Monotherapy	14572/827.54	0.16 (0.15, 0.16)	0.16 (0.15, 0.16)	0.24 (0.24, 0.25)	
Polytherapy	15375/1950.52	0.09 (0.08, 0.09)	0.09 (0.08, 0.09)	0.18 (0.18, 0.19)	
**Stratified analysis by sex**					0.02
**Men (N = 235,887)**					
Non-drug	24672/180.90	Reference	Reference	Reference	
Non-antipsychotic	677/28.18	0.18 (0.16, 0.19)	0.18 (0.16, 0.19)	0.23 (0.21, 0.25)	
Monotherapy	8284/440.78	0.16 (0.16, 0.17)	0.16 (0.16, 0.17)	0.25 (0.24, 0.26)	
Polytherapy	8600/1061.21	0.09 (0.09, 0.09)	0.09 (0.09, 0.09)	0.19 (0.18, 0.19)	
**Women (N = 199,929)**					
Non-drug	17682/126.41	Reference	Reference	Reference	
Non-antipsychotic	549/26.46	0.15 (0.14, 0.17)	0.16 (0.14, 0.17)	0.21 (0.19, 0.22)	
Monotherapy	6288/386.76	0.15 (0.14, 0.15)	0.15 (0.14, 0.15)	0.23 (0.23, 0.24)	
Polytherapy	6775/889.31	0.08 (0.08, 0.09)	0.08 (0.08, 0.09)	0.18 (0.18, 0.19)	
**Stratified analysis by age**					<0.001
**18−64 years (N = 393,034)**					
Non-drug	27026/239.92	Reference	Reference	Reference	
Non-antipsychotic	742/46.38	0.13 (0.12, 0.14)	0.13 (0.12, 0.14)	0.18 (0.17, 0.19)	
Monotherapy	9933/754.66	0.12 (0.12, 0.12)	0.12 (0.12, 0.12)	0.19 (0.19, 0.20)	
Polytherapy	12573/1882.03	0.07 (0.07, 0.07)	0.07 (0.07, 0.07)	0.16 (0.15, 0.16)	
**≥65 years (N = 42782)**					
Non-drug	15328/67.39	Reference	Reference	Reference	
Non-antipsychotic	484/8.26	0.25 (0.23, 0.27)	0.25 (0.23, 0.28)	0.31 (0.28, 0.34)	
Monotherapy	4639/72.87	0.30 (0.29, 0.31)	0.30 (0.29, 0.31)	0.42 (0.40, 0.43)	
Polytherapy	2802/68.49	0.20 (0.19, 0.21)	0.20 (0.19, 0.21)	0.33 (0.32, 0.35)	
**Stratified analysis by intensive mental health management**					<0.001
**No (N = 181,671)**					
Non-drug	41994/254.92	Reference	Reference	Reference	
Non-antipsychotic	1090/30.93	0.21 (0.19, 0.22)	0.21 (0.19, 0.22)	0.21 (0.20, 0.23)	
Monotherapy	12410/357.45	0.23 (0.23, 0.23)	0.23 (0.22, 0.23)	0.23 (0.23, 0.24)	
Polytherapy	11045/482.00	0.17 (0.17, 0.18)	0.17 (0.17, 0.17)	0.17 (0.17, 0.18)	
**Yes (N = 254,145)**					
Non-drug	360/52.40	Reference	Reference	Reference	
Non-antipsychotic	136/23.71	0.75 (0.61, 0.91)	0.77 (0.63, 0.93)	0.76 (0.63, 0.93)	
Monotherapy	2162/470.08	0.79 (0.70, 0.88)	0.82 (0.73, 0.91)	0.82 (0.73, 0.91)	
Polytherapy	4330/1468.52	0.74 (0.66, 0.82)	0.77 (0.69, 0.86)	0.77 (0.69, 0.86)	
**Stratified analysis by region of residence**					<0.001
**Rural (N = 178,048)**					
Non-drug	24762/166.34	Reference	Reference	Reference	
Non-antipsychotic	473/23.09	0.14 (0.13, 0.16)	0.14 (0.13, 0.16)	0.19 (0.17, 0.20)	
Monotherapy	5637/341.54	0.13 (0.13, 0.14)	0.14 (0.13, 0.14)	0.21 (0.20, 0.22)	
Polytherapy	6018/748.51	0.08 (0.08, 0.08)	0.08 (0.07, 0.08)	0.17 (0.16, 0.17)	
**Urban (N = 257,768)**					
Non-drug	17592/140.97	Reference	Reference	Reference	
Non-antipsychotic	753/31.55	0.19 (0.18, 0.20)	0.19 (0.17, 0.20)	0.25 (0.24, 0.27)	
Monotherapy	8935/486.00	0.18 (0.17, 0.18)	0.18 (0.17, 0.18)	0.28 (0.27, 0.29)	
Polytherapy	9357/1202.01	0.10 (0.09, 0.10)	0.10 (0.09, 0.10)	0.21 (0.20, 0.22)	

Cox proportional hazards regression models were used to estimate hazard ratios (HRs) with 95% confidence intervals (CIs).

Model 1: Adjusted for age (continuous) and sex (men or women).

Model 2: Additionally adjusted for educational attainment (continuous), marital status (married or unmarried), poverty level (yes or no) and region of residence (rural or urban).

Model 3: Further adjusted for number of comorbid conditions (0, 1–2 or ≥3), frequency of suicide attempts or self-harm episodes (0, 1–2 or ≥3), presence of positive symptoms (yes or no), presence of negative symptoms (yes or no) and intensive mental health management (yes or no).

P for interaction was derived from likelihood ratio tests comparing model 3 with and without interaction terms.

PY, person-years.

Stratified analyses revealed significant interactions between antipsychotic treatments and sex (*P* for interaction = 0.02), age (*P* for interaction < 0.001), schizophrenia severity as indicated by intensive mental health management (*P* for interaction < 0.001) and region of residence (*P* for interaction < 0.001). Specifically, greater benefits from antipsychotic treatments were observed among patients who were women, younger, had less severe schizophrenia or resided in rural areas (Table 2).

### Mortality risk by specific medications among patients receiving antipsychotic monotherapy

As shown in Table 3, FGAs had slightly higher crude mortality rates (19.81 per 1,000 PY) than SGAs (17.15 per 1,000 PY). After multivariable adjustment, however, both FGAs (aHRs: 0.26; 95% CI: 0.25–0.27) and SGAs (aHR: 0.26; 95% CI: 0.25–0.26) exhibited comparably reduced mortality risks relative to the non-drug group. Among FGAs, adjusted HRs ranged from 0.22 (95% CI: 0.20–0.25) for penfluridol to 0.25 (95% CI: 0.24–0.26) for perphenazine. For SGAs, adjusted HRs ranged from 0.19 (95% CI: 0.14–0.26) for ziprasidone to 0.35 (95% CI: 0.31–0.39) for amisulpride.

**Table 3.** Association between specific antipsychotic medication and mortality risk among schizophrenia patients receiving monotherapy Table 3 long description.

Antipsychotic group	Deaths/1000 PY	Model 1	Model 2	Model 3
**Non-drug**	42354/307.31	Reference	Reference	Reference
**FGAs**	2652/133.85	0.16 (0.15, 0.17)	0.16 (0.15, 0.17)	0.26 (0.25, 0.27)
Perphenazine	1638/78.73	0.15 (0.15, 0.16)	0.15 (0.14, 0.16)	0.25 (0.24, 0.26)
Chlorpromazine	450/20.41	0.17 (0.15, 0.18)	0.17 (0.15, 0.19)	0.24 (0.22, 0.26)
Sulpiride	523/32.74	0.14 (0.13, 0.15)	0.14 (0.13, 0.15)	0.24 (0.22, 0.26)
Penfluridol	303/16.12	0.14 (0.12, 0.16)	0.14 (0.12, 0.15)	0.22 (0.20, 0.25)
**SGAs**	11617/677.56	0.16 (0.16, 0.16)	0.16 (0.16, 0.17)	0.26 (0.25, 0.26)
Amisulpride	263/14.84	0.18 (0.16, 0.21)	0.19 (0.17, 0.22)	0.35 (0.31, 0.39)
Perospirone	58/3.52	0.16 (0.12, 0.20)	0.16 (0.13, 0.21)	0.33 (0.26, 0.43)
Quetiapine	520/28.04	0.16 (0.15, 0.18)	0.17 (0.15, 0.18)	0.28 (0.26, 0.31)
Olanzapine	2190/127.26	0.16 (0.15, 0.17)	0.17 (0.16, 0.17)	0.27 (0.26, 0.28)
Clozapine	3417/184.61	0.16 (0.15, 0.17)	0.16 (0.15, 0.16)	0.25 (0.24, 0.26)
Risperidone	4762/280.78	0.15 (0.14, 0.15)	0.15 (0.14, 0.15)	0.24 (0.23, 0.25)
Aripiprazole	343/31.90	0.11 (0.10, 0.12)	0.12 (0.11, 0.13)	0.20 (0.18, 0.22)
Ziprasidone	42/3.95	0.11 (0.08, 0.15)	0.12 (0.09, 0.16)	0.19 (0.14, 0.26)

Cox proportional hazards regression models were used to estimate hazard ratios (HRs) with 95% confidence intervals (CIs).

Model 1: Adjusted for age (continuous) and sex (men or women).

Model 2: Additionally adjusted for educational attainment (continuous), marital status (married or unmarried), poverty level (yes or no) and region of residence (rural or urban).

Model 3: Further adjusted for number of comorbid conditions (0, 1–2 or ≥3), frequency of suicide attempts or self-harm episodes (0, 1–2 or ≥3), presence of positive symptoms (yes or no), presence of negative symptoms (yes or no) and intensive mental health management (yes or no).

PY, person-years.

**Figure 2.**
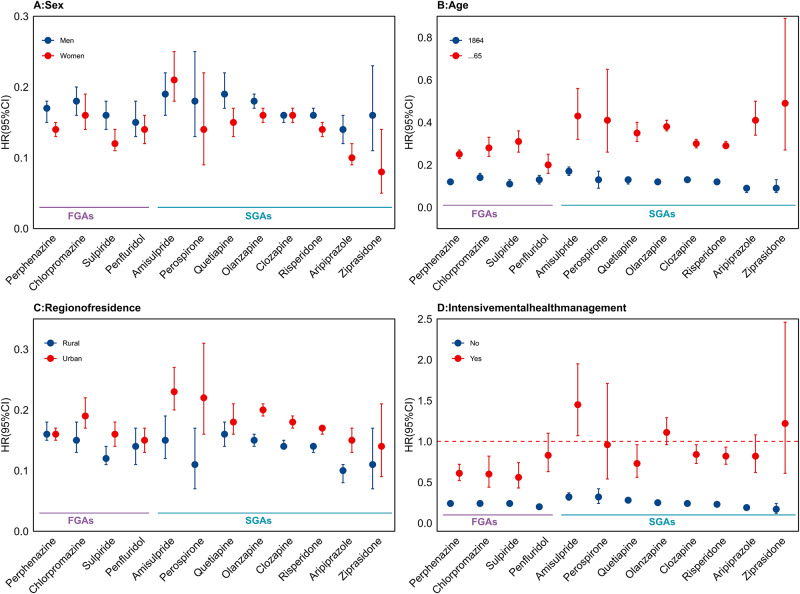
Stratified analyses of the associations between antipsychotic prescription and mortality risk among schizophrenia patients receiving monotherapy. Hazard ratios (HRs) and corresponding 95% confidence intervals (CIs) for all-cause mortality associated with antipsychotic treatments were calculated using a Cox proportional hazards model with the non-drug group as the reference category. HRs were adjusted for demographic characteristics, comorbid conditions, severity of schizophrenia and intensive mental health management. Analyses are stratified by (a) sex (men vs. women), (b) region of residence (urban vs. rural), (c) age group (<65 vs. ≥65 years) and (d) intensive mental health management (yes vs. no). Figure 2 long description.

Stratified analyses indicated that the associations between monotherapy and reduced mortality risk were more pronounced among schizophrenia patients who were women, younger, resided in rural areas and had less severe illness. Additionally, we observed that penfluridol, amisulpride, perospirone, olanzapine, aripiprazole and ziprasidone did not demonstrate significantly lower mortality risks compared with the non-drug group among patients with severe schizophrenia, despite significantly lower risks being observed among those with less severe illness (Fig. 2).

### Mortality risk associated with antipsychotic polytherapy combinations

**Figure 3.**
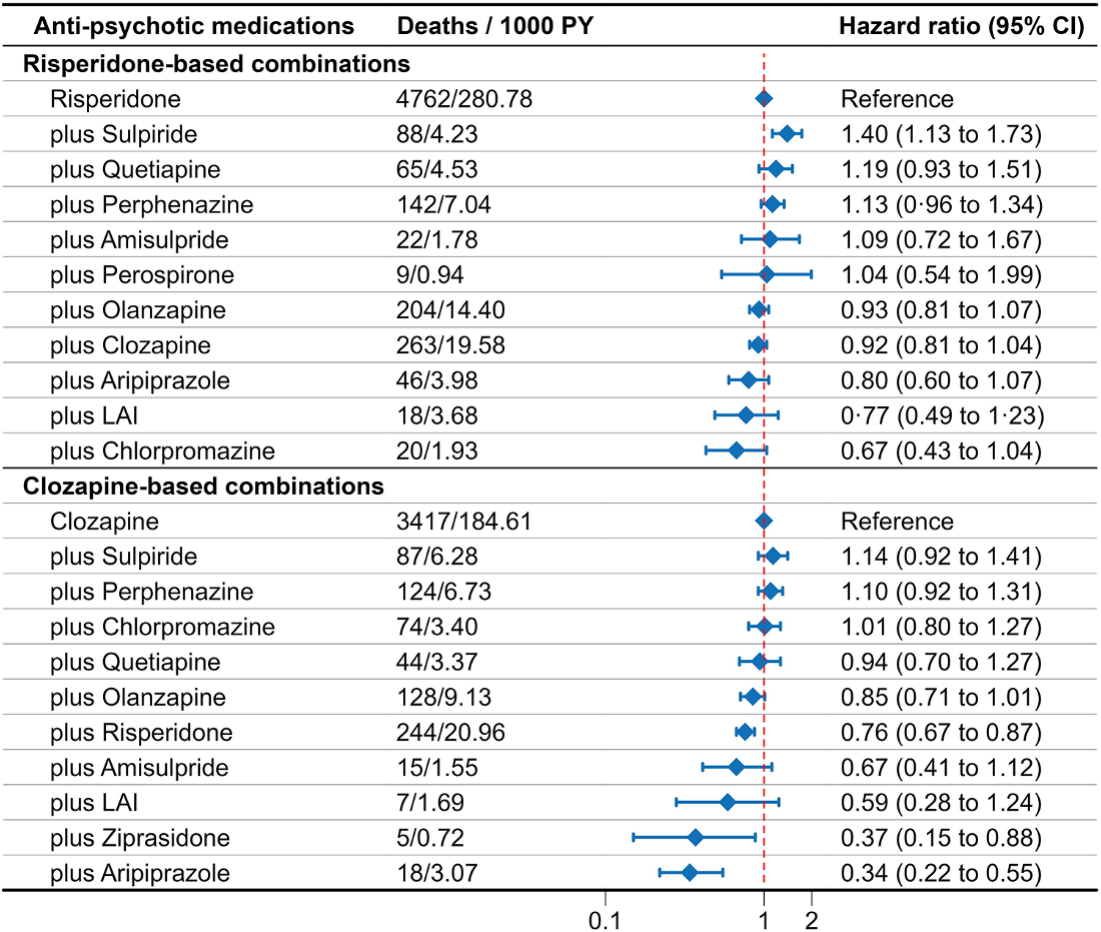
Associations between antipsychotic polytherapy combinations and mortality risk among schizophrenia patients receiving polytherapy. Cox proportional hazards regression models were used to estimate hazard ratios (HRs) and their corresponding 95% confidence intervals (CIs). PY, person-years. Adjusted HRs were controlled for age (continuous), sex (men or women), educational attainment (continuous), marital status (married or unmarried), poverty level (yes or no), region of residence (rural or urban), number of comorbid conditions (0, 1–2 or ≥3), frequency of suicide attempts or self-harm episodes (0, 1–2 or ≥3), presence of positive symptoms (yes or no), presence of negative symptoms (yes or no) and intensive mental health management (yes or no). Figure 3 long description.

As shown in Fig. 1f, among the 11.2 million polytherapy prescriptions, clozapine- and risperidone-based medication combinations were the most frequently prescribed in our cohort. We evaluated the associations between these medication combinations and mortality risk, using clozapine and risperidone monotherapies as references, respectively (Fig. 3). For risperidone-based combinations, most exhibited mortality risks comparable to risperidone monotherapy, except for risperidone-sulpiride, which was associated with a significantly increased mortality risk (HR: 1.40; 95% CI: 1.13–1.73). For clozapine-based combinations, most also demonstrated similar mortality risks compared to clozapine monotherapy; however, the combinations clozapine-risperidone (HR: 0.76; 95% CI: 0.67–0.87), clozapine-ziprasidone (HR: 0.37; 95% CI: 0.15–0.88) and clozapine-aripiprazole (HR: 0.34; 95% CI: 0.22–0.55) were significantly associated with reduced mortality risk.

### Sensitivity analyses

Excluding patients with follow-up durations shorter than 180 days did not materially alter the results (Supplementary Table 2). Similarly, when patients who entered the cohort prior to 2018 were excluded, our findings remained robust, although the strength of the associations between antipsychotic treatment and mortality risk was somewhat attenuated (Supplementary Table 3). Additional analyses that distinguished physical from psychiatric comorbidities produced results comparable to the primary models, with no meaningful change in the associations between treatment patterns and all-cause mortality (Supplementary Table 4).

## Discussion

This large-scale, real-world observational cohort study of 435,816 schizophrenia patients in Guangdong Province, China, offers valuable insights into antipsychotic prescribing patterns and their association with mortality risk. Over half of the patients received antipsychotic polytherapy during the follow-up period, whereas approximately one-quarter received monotherapy. Risperidone, olanzapine and clozapine were the most frequently prescribed antipsychotics. Notably, monotherapy, polytherapy and non-antipsychotic treatments were all associated with a significant reduction in mortality risk compared to the non-drug group. Among the monotherapies, both FGAs and SGAs exhibited comparably reduced mortality risks relative to the non-drug group. Most of the antipsychotic polytherapy performed as well as or better than monotherapy, although certain combinations, such as risperidone-sulpiride, increased mortality risk. Subgroup analyses revealed greater treatment benefits among women, younger patients, those with less severe schizophrenia and rural residents, highlighting the importance of personalized treatment strategies.

The prevalence of antipsychotic polytherapy in our study (57.6%) is notably higher than that reported globally and in previous Chinese studies. A systematic review estimated a global median polypharmacy rate of 19.6%, with higher rates in Asia compared to North America and Oceania (Gallego *et al.*, 2012). In China, previous studies have reported polypharmacy rates ranging from 12% to 34% (Li *et al.*, 2015; Qiu *et al.*, 2018), suggesting that our findings may reflect regional variations, differences in clinical practices or study methodologies. For instance, a study comparing China and Japan found a polypharmacy rate of 12.7% in China, possibly due to a stricter definition requiring medications to overlap for at least 60 days (Qiu *et al.*, 2018). In contrast, countries like Singapore have reported even higher rates (71.7%) (Sim *et al.*, 2004), while the rate in the U.S. was reported to be 16% (Gallego *et al.*, 2012).

Regarding overall treatment rates, approximately 85% of schizophrenia patients in our cohort received antipsychotic treatment, higher than previous findings from a meta-analysis estimating a 73% treatment rate (Qi *et al.*, 2020). This rate is comparable to that in developed countries, such as the U.S., where 84% of schizophrenia patients receive antipsychotics (Fitch *et al.*, 2014). The lower utilization of LAIs in China compared to developed countries remains a significant barrier, potentially due to cost and accessibility issues (Tang *et al.*, 2020). These differences underscore the need for strategies to enhance access to effective treatments like LAIs to close the global treatment gap.

Our study found no significant difference in mortality risk between FGAs and SGAs after adjusting for confounding factors, suggesting that both classes are equally effective in reducing mortality when compared to untreated patients. This finding is consistent with some prior research, such as a nationwide cohort study that found the initial mortality advantage of SGAs diminished after adjusting for prescribing patterns (Chen *et al.*, 2015). However, a systematic review suggested that SGAs might confer a survival advantage, possibly due to better tolerability and adherence (Jia *et al.*, 2022). Among specific monotherapies, clozapine did not demonstrate a distinct reduction in mortality risk in our Chinese cohort, contrasting with European studies that highlight its superior efficacy (Tiihonen *et al.*, 2009). This discrepancy may be attributed to ethnic-specific pharmacogenomic factors, particularly the higher prevalence of HLA-B alleles associated with clozapine-induced agranulocytosis (CIAG) in Asian populations, which increases the risk of infection-related mortality (Islam *et al.*, 2022).

The impact of antipsychotic polytherapy on mortality remains a contentious issue. For instance, a Hungarian study reported a 39% reduction in treatment failure risk with polypharmacy compared to monotherapy (Lähteenvuo and Tiihonen, 2021). However, other research has found no association or even increased mortality with polypharmacy, particularly when multiple antipsychotics are used concurrently (Baandrup *et al.*, 2010). In our cohort, most polytherapy combinations showed comparable or superior mortality risk reduction compared to monotherapy, although certain combinations, such as risperidone-sulpiride, were associated with increased risk.

These findings should not be interpreted as evidence of the superiority of polytherapy. Rather, they are likely to reflect differences in patient characteristics, treatment trajectories and clinical decision-making. Notably, the polytherapy category in this study encompassed both concurrent combination therapy and sequential switching between antipsychotics, which represent distinct clinical scenarios. Concurrent polytherapy is often reserved for more severe or treatment-resistant cases, whereas sequential switching typically reflects suboptimal response or intolerance to prior treatment. Such heterogeneity may obscure important differences in prognosis across treatment patterns and complicate interpretation.

Moreover, the observed associations for polytherapy may partly reflect factors beyond pharmacological effects. Patients receiving polytherapy may have more frequent clinical contact, closer monitoring and greater engagement with healthcare services, which could independently contribute to improved outcomes. These factors are not fully captured in registry data and may partially explain the observed associations.

Importantly, current clinical guidelines recommend antipsychotic monotherapy as the standard of care, with polytherapy reserved for specific clinical indications, such as treatment-resistant schizophrenia. Therefore, our findings should not be interpreted as supporting the routine use of antipsychotic polypharmacy. Given the potential risks associated with polytherapy, including increased adverse effects and drug interactions, caution is warranted. Further randomized controlled trials are needed to clarify the safety and efficacy of specific polytherapy strategies.

Our study identified greater treatment benefits among specific subgroups, including women, younger patients, those with less severe schizophrenia and rural residents. Women exhibited significantly lower mortality risks, consistent with a meta-analysis showing larger mean symptom reductions in women compared to men (number needed to treat: 6.9 for women vs. 9.4 for men) (Storosum *et al.*, 2023). This may be attributed to hormonal influences, such as the antidopaminergic effects of oestrogen, or better medication adherence. Younger patients benefited more likely due to fewer age-related comorbidities, such as cardiovascular disease and diabetes, which increase mortality risk in older adults (Tamargo *et al.*, 2022). Although younger age at onset is often associated with poorer prognosis, our findings suggest that current age, rather than onset age, drives treatment response. Patients with less severe schizophrenia showed greater treatment benefits, as milder forms of the illness tend to be more responsive to pharmacotherapy (Sampogna *et al.*, 2023). Rural residents also experienced greater benefits, possibly due to stronger social support networks, lower stress levels or differences in healthcare delivery compared to urban settings (Yang *et al.*, 2022). While limited data on rural-urban differences suggest that access to care may influence treatment outcomes, further research is needed to explore this. These findings underscore the importance of tailoring treatment to individual patient characteristics to maximize benefits.

The study’s strengths include its large, representative sample drawn from a comprehensive provincial mental health registry, extensive follow-up duration and robust comparative analysis of various antipsychotic regimens. These factors enhance the generalizability and reliability of our findings for clinical practice and policymaking. However, several limitations should be acknowledged. First, despite adjustment for measured confounders, residual confounding cannot be excluded. Unmeasured factors – such as socioeconomic status, healthcare access, disease severity, health-seeking behaviour and social support – may differ between treated and untreated patients and partly explain the observed associations. Second, incomplete or imperfect medication records may have introduced exposure misclassification, potentially underestimating mortality in the non-drug group. In addition, treatment exposure was defined over the entire follow-up period rather than as time-varying, which may introduce misclassification and survivorship bias, particularly for polytherapy, potentially exaggerating protective associations. Third, the outcome was limited to all-cause mortality, as cause-specific mortality data were unavailable, restricting insight into underlying mechanisms. Fourth, the non-drug group differed substantially from treated patients at baseline and likely represented a more vulnerable population with poorer access to care and lower treatment engagement; thus, comparisons using this group as the reference may overestimate treatment-associated risk reductions. Finally, treatment patterns may reflect underlying patient characteristics and clinical decision-making. For example, individuals in the non-drug group may have limited access to care or low adherence, while those receiving non-antipsychotic treatments may have medication intolerance or comorbid conditions. As these factors are not fully captured in the registry, they may contribute to the observed differences and should be considered when interpreting the findings.

## Conclusion

This population-based cohort study provides robust real-world evidence on antipsychotic treatment patterns and their association with mortality among patients with schizophrenia in China, although causal inference remains limited by the observational design. Both first- and second-generation antipsychotics were associated with reduced mortality risk, with no significant difference between them after adjustment. Polytherapy regimens showed mortality risks comparable to, or in some cases lower than, those observed with monotherapy; however, these findings should be interpreted cautiously and require confirmation in future studies. Notably, certain combinations, such as risperidone–sulpiride, were associated with increased mortality, underscoring the importance of careful drug selection. Greater benefits were observed among women, younger patients, those with less severe illness and rural residents, highlighting the potential value of individualized treatment strategies. Future research should clarify underlying mechanisms, refine treatment strategies – particularly regarding polytherapy – and address barriers to effective care, including access to long-acting injectable antipsychotics, to further reduce mortality in schizophrenia.

## Supporting information

10.1017/S204579602610078X.sm001 He et al. supplementary material He et al. supplementary material

## Data Availability

The datasets generated and/or analysed during the current study are not publicly available due to institutional policies but can be requested by contacting the corresponding author via email. Any data sharing will require approval from the Academic Committee of Guangdong Provincial People’s Hospital to ensure compliance with ethical and regulatory guidelines.

## Supplementary material

The supplementary material for this article can be found at https://doi.org/10.1017/S204579602610078X.

## Table 1 Long description

Baseline characteristics are summarized for 435,816 people with schizophrenia overall and by treatment group: non-drug, non-antipsychotic medications, antipsychotic monotherapy, and antipsychotic polytherapy. Group sizes were 57,335 non-drug, 6,771 non-antipsychotic, 120,487 monotherapy, and 251,223 polytherapy. Median follow-up was longest in the non-antipsychotic group (8.3 years) and shortest in the non-drug group (4.5 years). Mortality differed strongly by group: deaths were 73.9% in the non-drug group versus 18.1% in non-antipsychotic, 12.1% in monotherapy, and 6.1% in polytherapy. Median age at cohort entry was oldest in the non-drug group (54.2 years) and youngest in polytherapy (39.7 years); median age at schizophrenia diagnosis followed a similar pattern (35 years in non-drug and non-antipsychotic versus 27 years in polytherapy). Sex distribution was similar across groups (about half men), and nearly all participants were Han ethnicity (about 99.4%). Polytherapy had the highest share receiving intensive mental health management (72.4%), compared with 53.0% in monotherapy and 9.9% in the non-drug group. Most participants had no recorded comorbid conditions (about 95% or more in each group) and most had no suicide attempts or self-harm episodes (about 93% to 97%). Differences between groups may reflect underlying clinical severity, age, and care patterns rather than treatment effects.

Navigate back to Table 1.

## Figure 1 Long description

Infographic presents data on antipsychotic prescriptions among schizophrenia patients. A) Bar chart showing overall prescriptions with 17.1 million records. Risperidone, clozapine and olanzapine are the most prescribed. B) Stacked area chart showing cumulative percentage of overall prescriptions from 2007 to 2020. C) Bar chart showing monotherapy prescriptions with 4.2 million records. Risperidone, clozapine and olanzapine are prominent. D) Stacked area chart showing cumulative percentage of monotherapy prescriptions from 2007 to 2020. E) Bar chart showing polytherapy prescriptions with 11.2 million records. Clozapine-based combinations are frequent. F) Network diagram illustrating connections between antipsychotics in polytherapy, highlighting clozapine, risperidone and sulpiride.

Navigate back to Figure 1.

## Figure 2 Long description

Four graphs display hazard ratios (HR) with confidence intervals for various antipsychotic drugs, categorized by sex, age, region of residence and health management. Each graph uses point estimates with error bars. Plot A: Sex - The x-axis lists drugs: Perphenazine, Fluphenazine, Haloperidol, Penfluridol, Amisulpride, Perospirone, Olanzapine, Aripiprazole, Ziprasidone. The y-axis shows HR from 0 to 0.3. Men and women are distinguished by markers. Key finding: Women generally have higher HRs than men across most drugs. Plot B: Age - The x-axis lists the same drugs. The y-axis shows HR from 0 to 0.8. Age groups less than 65 and 65 or older are distinguished by markers. Key finding: Older age group shows higher HRs for several drugs. Plot C: Region of residence - The x-axis lists the same drugs. The y-axis shows HR from 0 to 0.3. Rural and urban regions are distinguished by markers. Key finding: Urban residents have slightly higher HRs for some drugs. Plot D: Health management - The x-axis lists the same drugs. The y-axis shows HR from 0 to 2.5. Categories ′yes′ and ′no′ are distinguished by markers. A dashed line at HR=1 indicates no effect. Key finding: Lack of management is associated with higher HRs for certain drugs. Each plot distinguishes between FGAs (first-generation antipsychotics) and SGAs (second-generation antipsychotics) with a horizontal line. Notable outliers and peaks are observed in specific drugs across different categories.

Navigate back to Figure 2.

## Figure 3 Long description

Forest plot showing hazard ratios for antipsychotic medication combinations compared to monotherapy. The x-axis represents the hazard ratio on a log scale, with a null value at 1.0. The plot includes two sections: risperidone-based and clozapine-based combinations. Each combination is listed with deaths per 1000 person-years and corresponding hazard ratios with 95 percent confidence intervals. For risperidone-based combinations, risperidone-sulpiride shows a hazard ratio of 1.40 (95 percent CI: 1.13 to 1.73), indicating increased mortality risk. For clozapine-based combinations, clozapine-aripiprazole shows a hazard ratio of 0.34 (95 percent CI: 0.22 to 0.55), indicating reduced mortality risk. The reference groups are risperidone and clozapine monotherapies. Key data points include risperidone-sulpiride and clozapine-aripiprazole, with significant differences from the null line at 1.0.

Navigate back to Figure 3.

## Table 2 Long description

Mortality rates and adjusted hazard ratios compare schizophrenia patients by antipsychotic exposure: no drug, non-antipsychotic drugs, antipsychotic monotherapy, and antipsychotic polytherapy. In the full cohort of 435,816 people, all treated groups have lower adjusted mortality risk than the no-drug reference; in the most adjusted model, hazard ratios are 0.22 for non-antipsychotic drugs, 0.24 for monotherapy, and 0.18 for polytherapy. Sex-stratified results are similar, with polytherapy lowest in both men and women; interaction by sex is reported as 0.02. Age modifies the association: among ages 18 to 64 years, adjusted hazard ratios are 0.18 (non-antipsychotic), 0.19 (monotherapy), and 0.16 (polytherapy), while among ages 65 years and older they are higher at 0.31, 0.42, and 0.33, respectively; interaction by age is reported as less than 0.001. Intensive mental health management also modifies results: without intensive management, adjusted hazard ratios range from 0.17 to 0.23, whereas with intensive management they are closer to 0.76 to 0.82; interaction is reported as less than 0.001. Region shows a similar pattern, with lower hazard ratios in rural than urban settings and interaction reported as less than 0.001. Death counts per 1,000 person-years are provided for each group, but comparisons should rely on the adjusted hazard ratios because models account for demographic, socioeconomic, clinical factors, and care intensity.

Navigate back to Table 2.

## Table 3 Long description

Mortality among people with schizophrenia on antipsychotic monotherapy is summarized as deaths per 1,000 person-years and hazard ratios with confidence intervals across three progressively adjusted models, using the non-drug group as the reference. The non-drug group reports 42,354 deaths over 307.31 person-years. In the fully adjusted model, both first-generation and second-generation antipsychotic groups have similar hazard ratios around 0.26 compared with the non-drug reference. Among first-generation agents, fully adjusted hazard ratios range from about 0.22 for penfluridol to about 0.25 for perphenazine and about 0.24 for chlorpromazine and sulpiride. Among second-generation agents, the lowest fully adjusted hazard ratios are ziprasidone at about 0.19 and aripiprazole at about 0.20, while amisulpride and perospirone are higher at about 0.35 and 0.33. Commonly used second-generation drugs cluster near the mid range, including risperidone about 0.24, clozapine about 0.25, olanzapine about 0.27, and quetiapine about 0.28. Interpretation should note that hazard ratios change after additional adjustment, and some drugs have relatively few deaths and person-years, which can make estimates less precise.

Navigate back to Table 3.
