# Supplementary material for: Real-world antipsychotic prescribing patterns and mortality in schizophrenia: a registry cohort study from China
Source: Epidemiol Psychiatr Sci. 2026 Aug 4;35:e46. doi: 10.1017/S204579602610078X (PMC13435155; doi:10.1017/S204579602610078X)
Supplement: Supplementary file 1 [file S204579602610078Xsup001.docx]

**Real-world Antipsychotic Prescribing Patterns and Mortality in Schizophrenia: A National Registry Cohort Study from China**

**Supplementary Table 1. List of antipsychotics used in the study**

**The oral or injectable first-generation antipsychotics (FGAs)**

Perphenazine

Sulpiride

Chlorpromazine

Penfluridol

Haloperidol

Fluphenazine decanoate

Haloperidol decanoate

Pipotiazine palmitate

Loxapine

Chlorprothixene

Thioridazine

Cloxapine

Chlorothiazide Hydrochloride

Pymetrozine

Trifluoperazine

**The oral or injectable second-generation antipsychotics (SGAs)**

Clozapine

Risperidone

Olanzapine

Ziprasidone

Perospirone

Aripiprazole

Quetiapine

Paliperidone

Amisulpride

Thiabery

Lurasidone-hydrochloride

Blonanserin

**long-acting injectables (LAIs)**

Paliperidone LAI

Haloperidol LAI

Perphenazine LAI

Risperidone LAI

Flupentixol decanoate LAI

FGAs: first-generation antipsychotics; SGAs: second-generation antipsychotics; LAIs:

long-acting injectables

**Supplementary Table 2. Sensitivity analysis: Association between antipsychotic prescription and mortality risk, excluding patients with follow-up duration shorter than 180 days**

| **Antipsychotic group** | **Deaths / 1000 PY** | **Model 1** | **Model 2** | **Model 4** |
| --- | --- | --- | --- | --- |
| Non-drug | 39836/306.78 | Reference | Reference | Reference |
| Non-antipsychotic | 1187/54.60 | 0.17 (0.16, 0.18) | 0.17 (0.16, 0.18) | 0.22 (0.21, 0.23) |
| Monotherapy | 13937/826.18 | 0.16 (0.15, 0.16) | 0.16 (0.15, 0.16) | 0.24 (0.24, 0.25) |
| Polyphtherapy | 15065/1949.48 | 0.09 (0.09, 0.09) | 0.09 (0.09, 0.09) | 0.19 (0.18, 0.19) |
| Non-drug | 39836/306.78 | Reference | Reference | Reference |
| FGAs | 2569/133.76 | 0.16 (0.16, 0.17) | 0.16 (0.15, 0.17) | 0.26 (0.25, 0.27) |
| Chlorpromazine | 448/20.40 | 0.17 (0.16, 0.19) | 0.17 (0.16, 0.19) | 0.25 (0.22, 0.27) |
| Perphenazine | 1580/78.68 | 0.15 (0.15, 0.16) | 0.15 (0.14, 0.15) | 0.25 (0.24, 0.26) |
| Penfluridol | 303/16.12 | 0.14 (0.13, 0.16) | 0.14 (0.13, 0.16) | 0.23 (0.20, 0.25) |
| Sulpiride | 502/32.70 | 0.14 (0.13, 0.15) | 0.14 (0.13, 0.15) | 0.24 (0.22, 0.26) |
| SGAs | 11065/676.30 | 0.16 (0.16, 0.16) | 0.16 (0.16, 0.17) | 0.26 (0.25, 0.26) |
| Amisulpride | 240/14.79 | 0.18 (0.16, 0.20) | 0.19 (0.17, 0.21) | 0.34 (0.30, 0.38) |
| Olanzapine | 2022/126.82 | 0.16 (0.15, 0.16) | 0.16 (0.16, 0.17) | 0.26 (0.25, 0.27) |
| Quetiapine | 483/27.94 | 0.16 (0.14, 0.17) | 0.16 (0.15, 0.18) | 0.28 (0.25, 0.30) |
| Perospirone | 47/3.51 | 0.14 (0.10, 0.18) | 0.14 (0.11, 0.19) | 0.29 (0.22, 0.39) |
| Clozapine | 3361/184.52 | 0.16 (0.16, 0.17) | 0.16 (0.15, 0.17) | 0.25 (0.25, 0.26) |
| Risperidone | 4540/280.31 | 0.15 (0.14, 0.15) | 0.15 (0.14, 0.15) | 0.24 (0.23, 0.24) |
| Aripiprazole | 317/31.83 | 0.11 (0.10, 0.12) | 0.11 (0.10, 0.13) | 0.19 (0.17, 0.22) |
| Ziprasidone | 37/3.94 | 0.11 (0.08, 0.15) | 0.11 (0.08, 0.16) | 0.18 (0.13, 0.25) |

Cox proportional hazards regression models were used to estimate hazard ratios (HRs) with 95% confidence intervals (CIs).

Model 1: Adjusted for age (continuous) and sex (men or women).

Model 2: Additionally adjusted for educational attainment (continuous), marital status (married or unmarried), poverty level (yes or no), and region of residence (rural or urban).

Model 3: Further adjusted for number of comorbid conditions (0, 1–2, or ≥3), frequence of suicide attempts or self-harm episodes (0, 1–2, or ≥3), presence of positive symptoms (yes or no), presence of negative symptoms (yes or no), and intensive mental health management (yes or no).

PY, person-years.

**Supplementary** **Table 3. Sensitivity analysis: Association between antipsychotic prescription and mortality risk, excluding patients enrolled before 2018**

| **Antipsychotic group** | **Deaths / 1000 PY** | **Model 1** | **Model 2** | **Model 3** |
| --- | --- | --- | --- | --- |
| Non-drug | 727/9.07 | Reference | Reference | Reference |
| Non-antipsychotic | 183/4.77 | 0.45 (0.38, 0.53) | 0.46 (0.39, 0.54) | 0.56 (0.48, 0.66) |
| Monotherapy | 3617/129.86 | 0.48 (0.44, 0.52) | 0.49 (0.45, 0.53) | 0.66 (0.61, 0.71) |
| Polyphtherapy | 3486/297.94 | 0.30 (0.27, 0.32) | 0.30 (0.28, 0.33) | 0.49 (0.45, 0.54) |
| Nondrug | 727/9.07 | Reference | Reference | Reference |
| FGAs | 462/11.22 | 0.56 (0.50, 0.63) | 0.56 (0.50, 0.63) | 0.70 (0.62, 0.79) |
| Chlorpromazine | 48/1.32 | 0.44 (0.33, 0.59) | 0.46 (0.34, 0.62) | 0.55 (0.41, 0.73) |
| Perphenazine | 319/5.94 | 0.61 (0.53, 0.69) | 0.58 (0.51, 0.66) | 0.75 (0.66, 0.86) |
| Penfluridol | 21/0.98 | 0.27 (0.18, 0.42) | 0.27 (0.17, 0.41) | 0.37 (0.24, 0.58) |
| Sulpiride | 88/3.72 | 0.38 (0.31, 0.48) | 0.39 (0.31, 0.48) | 0.53 (0.42, 0.66) |
| SGAs | 3134/117.66 | 0.52 (0.48, 0.57) | 0.53 (0.48, 0.57) | 0.69 (0.63, 0.74) |
| Amisulpride | 113/4.80 | 0.59 (0.48, 0.72) | 0.61 (0.50, 0.74) | 0.86 (0.71, 1.05) |
| Olanzapine | 900/33.21 | 0.47 (0.43, 0.52) | 0.49 (0.45, 0.54) | 0.68 (0.61, 0.75) |
| Quetiapine | 169/6.00 | 0.45 (0.38, 0.53) | 0.46 (0.39, 0.55) | 0.60 (0.51, 0.71) |
| Perospirone | 36/1.35 | 0.45 (0.32, 0.63) | 0.46 (0.33, 0.64) | 0.65 (0.46, 0.90) |
| Clozapine | 519/17.04 | 0.53 (0.47, 0.59) | 0.52 (0.46, 0.58) | 0.68 (0.60, 0.76) |
| Risperidone | 1241/46.64 | 0.47 (0.43, 0.52) | 0.47 (0.43, 0.51) | 0.63 (0.57, 0.69) |
| Aripiprazole | 125/6.75 | 0.42 (0.35, 0.51) | 0.44 (0.36, 0.53) | 0.61 (0.50, 0.73) |
| Ziprasidone | 19/1.04 | 0.41 (0.26, 0.65) | 0.43 (0.27, 0.68) | 0.50 (0.32, 0.80) |

Cox proportional hazards regression models were used to estimate hazard ratios (HRs) with 95% confidence intervals (CIs).

Model 1: Adjusted for age (continuous) and sex (men or women).

Model 2: Additionally adjusted for educational attainment (continuous), marital status (married or unmarried), poverty level (yes or no), and region of residence (rural or urban).

Model 3: Further adjusted for number of comorbid conditions (0, 1–2, or ≥3), frequence of suicide attempts or self-harm episodes (0, 1–2, or ≥3), presence of positive symptoms (yes or no), presence of negative symptoms (yes or no), and intensive mental health management (yes or no).

PY, person-years.

**Supplementary Table 4. Sensitivity analysis: Association of antipsychotic treatment with mortality risk after separating psychiatric and physical comorbidities.**

| **Antipsychotic group** | **Model 4** | **Model 5** |
| --- | --- | --- |
| **Total participants (N=435,816)** |  |  |
| Non-drug | Reference | Reference |
| Non-antipsychotic | 0.22 (0.21, 0.23) | 0.24 (0.23, 0.26) |
| Monotherapy | 0.24 (0.24, 0.25) | 0.27 (0.26, 0.27) |
| Polytherapy | 0.18 (0.18, 0.19) | 0.20 (0.20, 0.21) |

Cox proportional hazards regression models were used to estimate hazard ratios (HRs) with 95% confidence intervals (CIs).

Model 4: The model was adjusted for age (continuous), sex (men or women), educational attainment (continuous), marital status (married or unmarried), poverty level (yes or no), and region of residence (rural or urban), physical comorbidities (yes or no, frequency of suicide attempts or self-harm episodes (0, 1–2, or ≥3), presence of positive symptoms (yes or no), presence of negative symptoms (yes or no), and intensive mental health management (yes or no).

Model 5: The model was adjusted for age (continuous), sex (men or women), educational attainment (continuous), marital status (married or unmarried), poverty level (yes or no), and region of residence (rural or urban), psychiatric comorbidities (yes, no or unknow), frequency of suicide attempts or self-harm episodes (0, 1–2, or ≥3), presence of positive symptoms (yes or no), presence of negative symptoms (yes or no), and intensive mental health management (yes or no).
